# Anorexia nervosa through the lens of a severe and enduring experience: ‘*lost in a big world’*

**DOI:** 10.1186/s40337-023-00953-2

**Published:** 2024-01-22

**Authors:** Laura Kiely, Janet Conti, Phillipa Hay

**Affiliations:** 1 School of Medicine, Translational Health Research Institute, Western Sydney University, Sydney, Australia; 2 School of Psychology, Translational Health Research Institute, Western Sydney University, Sydney, Australia; 3 Mental Health Services, Camden and Campbelltown Hospitals, SWSLHD, Campbeltown, NSW 2560 Australia

**Keywords:** Lived-experience, Phenomenology, Visual art, Qualitative, Interpretative phenomenological analysis, Severe and enduring, Anorexia nervosa, Eating disorder, Terminal, Fatal, Death

## Abstract

**Background:**

Severe and enduring anorexia nervosa (SE-AN), is a serious and persistent illness, despite ‘state of the art’ treatment. Criteria have been theoretically proposed, but not tested, and may not adequately capture illness complexity, which potentially inhibits treatment refinements. The clinical reality of death as an outcome for some people who experience SE-AN (1 in 20) and broadening access to voluntary assisted dying, further complicates the field, which is undeveloped regarding more fundamental concepts such as nosology, treatment, recovery definitions and alternative conceptualisations of SE-AN. The present paper is in response to this and aims to build upon qualitative literature to enhance phenomenological understandings of fatal SE-AN.

**Method:**

A published book, being the legacy of a 32-year-old professional artist offers a rich account of a life lived with AN, for 18 years with continuous treatment. A polysemous narrative via the interrelationship between the languages of the artist’s words and visual art is translated via interpretative phenomenological analysis (IPA), offering rich insight into the SE-AN experience.

**Findings:**

The process of analysis induced three superordinate themes (1) Disappearing Self (2) Dialectical Dilemma (3) Death and Dying: Finding Meaning. Two cross cutting themes traversed these themes: (a) Colour and (b) Shifting Hope, where the former produced a visual representation via the ‘SE-AN Kaleidoscope’. Collectively the themes produce a concept of SE-AN, grounded in the data and depicted visually through the artist’s paintings.

**Conclusions:**

The picture of SE-AN revealed in the analysis extends upon conceptualisations of SE-AN, highlighting key processes which are thus far under explored. These factors are implicated in illness persistence eliciting opportunities for further research testing including diagnostic considerations and treatment directions. In SE-AN, distorted body image extends to a global distortion in the perception of self. Additional criteria for the severe and enduring stages of illness related to (1) self and identity processes (2) measures of ‘global impoverishment’ across life domains are proposed for consideration in the future testing of putative defining features of SE-AN.

**Supplementary Information:**

The online version contains supplementary material available at 10.1186/s40337-023-00953-2.

## Abstract (plain language summary)

Anorexia Nervosa (AN) is a serious and life-threatening illness. There is a need to understand more about why AN persists in becoming severe and enduring (SE-AN) for some people. One such way to build understanding of a condition is via the stories of those who experience it. This study utilizes a person’s unique artistic language to do so. This has the potential to generate new ideas about a condition, especially those that may be beyond words for some people and therefore progress classifications for SE-AN for research and treatment purposes. The artist in this study contributes a unique perspective offering new areas for potential research such as understudied complex psychological processes for example shame, dissociation, ‘self’, emotional literacy, and anorexia as an identity. Additionally, other factors to be considered in the assessment and classifications of long-term cases of AN as well as an alternative understanding of AN persistence is proposed, beyond the concept of ‘body image disturbance’. Alternative treatment approaches such as art therapy are indicated.

**Supplementary Information:**

The online version contains supplementary material available at 10.1186/s40337-023-00953-2.

## Abstract (highlights)

**Note: Contents of this paper may be distressing to people who have lost a loved one to an eating disorder** and the following website may provide access to support if any of the content of his paper causes the reader distress:

USA - NEDA https://www.nationaleatingdisorders.org/

UK BEAT https://www.beateatingdisorders.org.uk/

Australia Butterfly https://butterfly.org.au/ or Lifeline - which is an international organisation with local websites.

**Supplementary Information:**

The online version contains supplementary material available at 10.1186/s40337-023-00953-2.

## Background

Severe and enduring anorexia nervosa (SE-AN), first described in 1981 [1] as ‘chronic AN’, is a serious illness that persists beyond an ‘acceptable’ timeframe, despite ‘state of the art’ treatment [2]. AN endures in 20–50% of people [3–5], commensurate with profound psychosocial impairment [6] and higher mortality, carer and healthcare burden relative to schizophrenia and depression [7]. Despite this, inequities in funding for research and treatment persist [8]. Currently proposed criteria for SE-AN (e.g., Hay and Touyz, 2018 [9]) are theoretically based and include severity, duration and treatment exposure specifiers. However, applying such criteria in clinical or research contexts is problematic, including how severity is defined. Severity in SE-AN is most often conceptualized on a continuum model [10]. Whilst DSM-5 [11] applies Body Mass Index (BMI) as the primary measure of severity, functional impairment is not predicted by BMI [12, 13], nor does duration necessarily relate to severity [14] or treatment response [15, 16].

A systematic review of qualitative research on the experience of SE-AN has highlighted its complexity, including ego-syntonicity beyond the valuing of symptoms and as compatible with a self-view of worthlessness and as intrinsic to the self [17]. It continues largely unknown as to why, and for whom, AN progresses to SE-AN. Furthermore, the current ‘state of the art’ AN treatments have low engagement, 50% attrition [18] and high rates of relapse [19–21]. When treatment does not work, reasons cited by those with a lived experience include that their needs often have not been met [22] and for some, treatment is experienced as harmful or traumatic [23]. Nevertheless, one RCT for SE-AN has found that when a person-centred approach is adopted and treatments shift from the focus of reduction in ED symptoms to quality of life, that symptoms may yet improve [24].

These factors pose a challenge to theoretical conceptualizations of AN, which along with medicalized classifications [11, 25] inform the basis of diagnosis, treatments and recovery [26, 27].

Treatments for SE-AN in adults are ideally delivered in a person-centered way, within a continuum of care, that prioritizes the least restrictive option [28]. Outpatient, manualized psychological therapies (described in detail elsewhere e.g. NICE [29]) are the first treatment pathway and inpatient hospitalization is typically reserved for medical stabilization. Involuntary admissions are often part of the course in SE-AN [30]. Medications have limited evidence with mixed results for anti-depressants and anti-psychotics. Anxiolytics are underexplored and psychedelics e.g. psilocybin as well as other brain based therapies [31] are experimental in SE-AN, with preliminary evidence [6]. The efficacy and *safety* of repeated delivery of the same treatments over decades is not established and given that less than 63% of people with AN have achieved recovery after 22 years [4], ongoing care options are needed. Specific care for SE-AN is limited to the evidence of a single clinical trial [32] and by necessity, community-based, recovery orientated care is described, but seldom available [33]. Treatment direction is limited by chronic underfunding of ED [8] and decisions related to care are fraught for health care providers, patients and families [34].

Whilst there is unanimous agreement that AN is complex, this has not been translated into treatment [35, 36]. If SE-AN is considered as a progression from AN, then phenomenological understandings have scope to assist in the identification of persistent features immune to or not currently targeted in AN treatment. An early conceptualisation of AN as a ‘self-disorder’ by Bruch [37] has offered an aetiological perspective, that an under-developed sense of self persists to undermine identity formation in adolescence. *‘Pursuit of thinness’* is positioned as a manifestation of a ‘*defective self-concept’*, a ‘*camouflage*’ to conceal underlying problems of a *‘fundamental inadequacy’, 'inaccuracy in perception and language of bodily sensation’* and ‘*fear of inner emptiness*’ [37] (pp 4–9). Conceptualisations of self, have not been comprehensively considered within dominant approaches to AN, limited to a cognitive behavioural focus of self-perception [38–40]. This position is affirmed in a recent systematic review which highlighted that self-constructs such as low self-esteem, self-concept, self-worth and cognitive schemas fall short of an encompassing, nuanced and unified definition of ‘self’ as lost to AN [41]. Rather, the traditional focus is more cognitively based, where issues with cognitive inflexibility (‘set shifting’), poor central coherence, distorted core self-beliefs as related to AN, inform treatment targets [39, 40, 42]. It is notable that evidence is mixed for these constructs in AN e.g. perceptual flexibility [43, 44]. Marking a shift in ED treatments, a feature of the MANTRA model is constructing an ‘identity’ outside of AN [41]. Additionally, starvation is understood to biologically exacerbate rigidity and distort self-perception via neurobiological changes [45]. Thus, whilst the cognitive aspect is undoubtedly important in self-perception, this may lack the depth to fully elucidate putative mechanisms that more broadly interrupt sense of self, particularly within a chronic, unremitting trajectory. Consistent with our previous review, in which AN was positioned to take over and diminish the self, we purport that formative, developmental and relational aspects in constructing a sense of self have not been adequately considered in AN conceptualizations and treatments [17].

Extending upon these insights include the understanding that shame processes, existential crisis and difficulties ‘being’ in the body, manifest in self and identity disturbance as represented through AN [17]. In this sense, ‘body image disturbance’ is less literal and relates to a more comprehensive and nuanced global disturbance of a (dis)embodied self [46–49]. Where the cogent self represents integration of affective, bodily, cognitive, emotional, relational, and somatosensory aspects [49]. Zucker et al. [50] posit that bodily ‘hypersensitivity’ may predispose a disturbance in body experiences and perceptions, a position affirmed in recent longitudinal findings regarding extremes of sensitivity as pre-dating AN [51]. Thus, conceptualisations of ‘self’ in AN have a substantial theoretical history [37, 46, 52–55], firmly established in phenomenological syntheses of AN [41, 56–59] and SE-AN [17] and warrant further expansion and exploration beyond the current dominant paradigms.

The clinical reality of death as an outcome for some people who experience SE-AN (1 in 20) and broadening access to voluntary assisted dying and end of life care [60–62], further complicates the field. This is reported to risk hope for those who live this complex reality [62–64]. The field is in a critical place, prompting an urgent need for novel conceptualizations and psychotherapeutic innovations. Thus, tuning in to the voices of lived experience to phenomenologically understand SE-AN has scope to explore new ideas.

Few studies have (i) exclusively focused on the qualitative experiences of SE-AN; and (ii) differentiated participants whose AN experience is severe and enduring [17, 65]. The present paper is in response to this and aims to; explore the SE-AN experience from the perspective of an artist who depicted their journey (until death), in a series of paintings and textural interpretations of the art. We anticipate that as a result of this exploration, new ideas might be generated for SE-AN nosology and treatment for (a) testing in further empirical studies and (b) exploration in triangulated studies of people with SE-AN.

## Method

### Epistemology

As a reflexive, inductive method, Interpretative Phenomenological Analysis (IPA) is compatible with the epistemological position of this study’s research question, which seeks to understand a person’s experience of SE-AN to illuminate—what is SE-AN?

### Data

The material selected for this study relates to a published book [66], which offers a polysemous narrative via the interrelationship between the languages of words and visual art. The book was curated by the artist as their artistic legacy, rendering it a unique contribution to the phenomenological understanding of SE-AN.

### Materials: about the book

The book includes digital reproductions of 21 paintings, personally selected by a professional artist who lived with SE-AN. She interpreted the works, in what would become her final months of life, rendering it a unique contribution to the phenomenological understanding of SE-AN. The artist used a visual language to communicate their experience which they were then able to use as a basis to develop a verbal narrative via their interpretations. The artist relied on the paintings, as the primary medium of self-expression, which afforded their retrospective interpretation to text, for their book which constitutes the single case report for this research.

### Analysis

IPA is a methodological process commonly used in health research to explore how individuals make sense of their world [67]. This method is proposed as the most appropriate to gain an in-depth understanding of the artist’s lived experience of SE-AN. IPA is methodologically adaptable and grounded in theories of phenomenology, hermeneutics and ideography [68]. Its task is to interpret via hermeneutics the implicit in the most unfiltered way allowing for the novel, interesting and unexpected, such that it can then be translated to a common or shared psychological framework by denoting themes for broader application [69]. It is foremost idiographic in that it holds the individual’s experience as unique and within its own context, where it is central and unconcerned with fitting one experience with a collective view (nomothetic) or clouded by another’s preconceptions or ‘phenomenology’. The phenomenological aspect of IPA has foundations in Edmond Hurssel’s (1900–1970) philosophy, which focuses on ‘how’ a person experiences something as a composite of their conscious, pre-conscious and unconscious processes in the context of their broader experiential field. This forms a person’s ‘life-world’ [70], influencing the lens through which they experience life and give meaning to those experiences. Phenomenology can be described simply as ‘meaning making’. Given that art is a metaphorical depiction of lived experience, Ricoeur’s notion of ‘living metaphor’ [71] extended this phenomenological analysis particularly in the aim to bridge the artist’s lived experience through art and language with the scientific pursuit of taxonomy in understanding SE-AN.

It is acknowledged that the researcher(s) bring their own preconceptions and meanings which, in order to truly encounter another person’s experience with ‘fresh eyes’ they must reflect upon and set aside. This is achieved through ‘Epoché’ or bracketing [72] and is grounded in the analyst’s reflective statement (see below), where the researcher identifies aspects of their own experience that could obscure a fresh view—for example prior encounters with people with SE-AN. The process of the researcher interpreting the participant’s construction of their world, is referred to as a ‘hermeneutic’. In the case of this study, which utilizes a person’s interpretations of their visual representations through artwork, there is a double hermeneutic and multiple ‘hermeneutic circles’ (interpretative processes) are manifest [68] with potential to offer more complex interpretations and enrich the findings. We therefore extended the method to encompass an ‘expanded hermeneutic phenomenology’ [73]. The artworks were used as the basis for the artist to create a shared language, enhancing reflexivity, whereby the primary experience was expressed via emotion and the unconscious ‘*With still life, I usually work from life, but this image just came to my mind *[66]* p36*., via the artwork. We purport that the artist’s words are constructed from the images, the two are interrelated and one cannot exist without the other. Retrospective interpretation by the artist via text alongside the images, allowed for the researcher to remain closer to the artists own interpretations.

To address the research question, IPA was flexibly adapted as outlined above to produce a series of 13, systematic and rigorous steps, satisfying the methodological processes outlined by Smith and Osborn [68, 74]. The analysis proceeded across each of the 40 pages of the book, the first page of which is shared by way of example in Table 1**.** All interpreters repeatedly read the book and collaborated on their free text analysis of the text and paintings which facilitated several processes. Firstly, familiarization with the material as a whole, noting specific qualities of the words (see Table 1—step 2a) and paintings (see Table 1—step 2b), and then to distill the salient elements of importance to the experiencing person and gain a general sense of the person from their choice of words and artistic style. At this stage also, the personal impact of the material was debriefed, to distill aspects for bracketing. The book text was then summarized and paraphrased line by line in the analysers’ (LK) words, grounded in the artist’s dialogue (Table 1—step 3) and with reference to the relevant image. Here, each image was discussed between all the authors regarding size, colour, medium, subject, content, metaphor, ambience, or distinguishing features, which was later clustered and related back to the emergent themes. For example: the theme ‘loss of self’ manifested in a temporal flux in colour and definition in the artworks, which aligned with explorations of death (loss of self) and ‘hope’ through the re-authoring of self. The ‘dialectical dilemma’ was illustrated via recurrent use of subject e.g. banksias as a metaphor for AN, to convey a polarized ambience of the AN experience (e.g. torture v’s comfort v’s loss). In other cases, twin scenes were created, one with and without colour which powerfully conveyed the artist’s dual reality.

**Table 1:** Extracted example of IPA methodology (steps 2–5/13) and (steps 6–13)

2a. Free text analysis (all authors)	2b. Free text analysis paintings	Book text	Lines	3. Summarize/paraphrase book text line by line
The sea-shell as a metaphor for the AN. It offers dual function i.e. it provides satefy and protection from a large overwhelming world Yet is insurmountably heavy, imperfect, stifling eating disorder as a trap—impermeable nourishment cannot come in, disallowing growth and I can’t be seen	Title: Shell, Turnip and another shell 2013, oil on tile, 30 × 45 cm Interpreters’ comments (via discussion) Painting is heavy (ceramic), fragile and breakable Intricately rendered, delicate, highly detailed, beautiful, pale, feminine, distortion (shadows) Image: Two shells with turnip (food) between them	I am a **shell, pale** and **white** **Safe, protected**, **milked** of **colour** I **curl up** in a ball I want to **hide** **Heavy, bumpy, smooth** I **would like** to **shine**, but I **can’t** I am **empty** A creature inside the shell A little, **little thing lost** in a **big** world I don’t want to **disturb anything** or **anyone**	1–4 5–9 10–11 12–13 14–15	I am a pale white shell and inside it I feel safe and protected but I’m losing my colour I make myself small (curl up) and hide I don’t want to be seen my heavy, bumpy smooth, shell I can’t shine in the shell I feel empty, and I feel lost in the shell. Am I the shell or the creature? I'm little, a ‘thing’, I’m lost, the world is bigger than me—overwhelming hiding in my shell I don’t disturb anything or anyone. I’m safe

4. Translation—higher level abstraction to psychological framework (Gestalt, narrative, psychodynamic)	5. Preliminary themes—6. intersubjectivity part 1	7. Consolidated (groups) + 8. Constituent themes 9. Chronology	10. Clustering/Subordination * 13. Intersubjectivity part 2	
The seashells are a metaphor for the AN Metaphor as communication AN offers dual function—polarity/dialectic Safety and protection—Fear Ambivalence about engaging in ‘the world’ AN as ‘defence’: Stuck-ness Defense mechanism/contact interruption: insurmountably heavy, imperfect, stifling Defence: the artist—milked/robbed Self: as heavy Self: who am I—identity processes? Withdrawal and Disconnection Shame processes (being seen) Art as self True self projected **Painting** distorted self (shadows, hiding) versus true self (Intricately rendered, delicate, highly detailed, beautiful, pale, feminine) reflected in the artistic style	**Self—intra/interpsychic processes** Metaphor and painting as language Self-expression Sense of self Self colour/pale Fragility Size and weight—corporeality Identity Projection Shame deprivation physically, socially and emotionally social and emotional withdrawal isolation aloneness emptiness unworthiness/hiding/shame **Dialectic** Safety Protection Stuck immobilized **Hope(less-ness)** Hopeless–helpless	Metaphor Colour Self AN Dialectic Hope Embodiment Identity Inter-psychic processes (withdrawal) Intrapsychic processes (shame)	Disappearing Self: ‘*Lost in a big world’* Embodied intra-psychic processes: vulnerable self Colour Shifting Hope Dialectical Dilemma	

*Steps (1) reading of text, (11) Cross referencing and directory of steps was done by colour coding and (12) directory are omitted from this example

The hermeneutic then progressed to higher level abstraction to psychological themes e.g. ‘stuck-ness’, ‘metaphor’, ‘psychological defense’ adopting the theoretical language of the first author’s (LK) background. Given a range of perspectives of the researchers (gestalt (LK), narrative (JC), psychodynamic (PH)) we chose to confer on the preliminary themes at this point and found that each author had a language for similar themes e.g. ‘defense’ versus the Gestalt term ‘contact interruptions’.

The analysis process was completed in two parts. Initially, preliminary themes were developed (2021) and consistent with Braun and Clarke’s lecture [75] exploring good practice in thematic analysis, the authors returned to the preliminary themes many months later, which resulted in further refinements and insights. We then proceeded with the consolidation and clustering (steps 7–10), of both the words and paintings. We conferred for intersubjectivity again at this final stage by way of addressing the bias unique to this study whereby the artist was known to the first author. Consideration of the Critical Appraisal Skills Program CASP [76] checklist informed study design and rigour.

### Authors’ reflective statements

The phenomenological lens impacts the process of synthesis, informed by collective personal and professional life experiences of the researchers. Of relevance for this review—Laura Kiely (LK) has worked clinically with people experiencing eating disorders (ED), encompassing end stage care, as an accredited practising dietitian and, professionally registered psychotherapist/counsellor, specialising in Gestalt Therapy (GT) interventions. GT fits within an existential, relational paradigm incorporating phenomenology as integrated with body process, and this informed the theoretical framework for the analysis. The artist’s book was bequeathed to LK by the artist and her family to edit and publish posthumously so she was familiar with the artists story and took care not to bring in material to the analysis that was outside of that shared in the book. For example, LK knew the context in which some of the artworks arose. e.g. following hospitalisation and this was set aside. LK’s clinical and personal experience of the limitations of current paradigms, together with treatment inadequacies to support the complex care needs of SE-AN, provided the impetus for the doctoral research of which the present paper forms one chapter. Because of the personal impact of these limitations, she is invested in exploring alternative paradigms, which ultimately paved the path towards research to influence change—a choice-fullness afforded by multiple privileges. Many layers of supervision—research supervision, clinical, peer and group supervision supported the author to at times put aside and and other times expand assumptions. So as to remain true to the phenomenological method and ensure gaps were addressed in the hermeneutic process with additional triangulation between researchers.

Janet Conti (JC) is a Clinical Psychologist and academic in Clinical Psychology who started her work with people who experience EDs as a dietitian. Her research and clinical work are informed by the paradigm of narrative therapy [77] and seek to prioritize the voice of the experiencing person to inform the development of a broader range of ED treatment interventions that have scope to be flexibly tailored to the needs and preferences of the experiencing person and their family. Phillipa Hay (PH) is a clinical academic Psychiatrist formally trained first in psychodynamic psychotherapy and then in cognitive behaviour therapy. She has experience of caring for many people with SE-AN in general hospitals and outpatient private practice setting, was a lead investigator on a SE-AN clinical trial [24], and lead author on Australian guidelines [78] endorsing the need for new person centered and flexible approaches in care.

### Ethics and quality consideration

The material was volunteered as the artist’s parting legacy to the first author (LK), to give hope to those with a lived AN experience and inspire ideas for treatment innovations. The artist endorsed the book manuscript prior to her untimely death, and it was posthumously published by LK (invited editor) on behalf of the artist and her family. These were unique ethical considerations as part of this study, which were navigated with input from an ethics committee and the artist's family. This enabled sensitive progression of the research where both saw this undertaking as a way to be respectful toward the person who died. The study received ethics approval from the Human Research Ethics Committee at Western Sydney University (HREC Approval number: H15036).

### Thematic representation: SE-AN Kaleidoscope

**Fig. 1:**
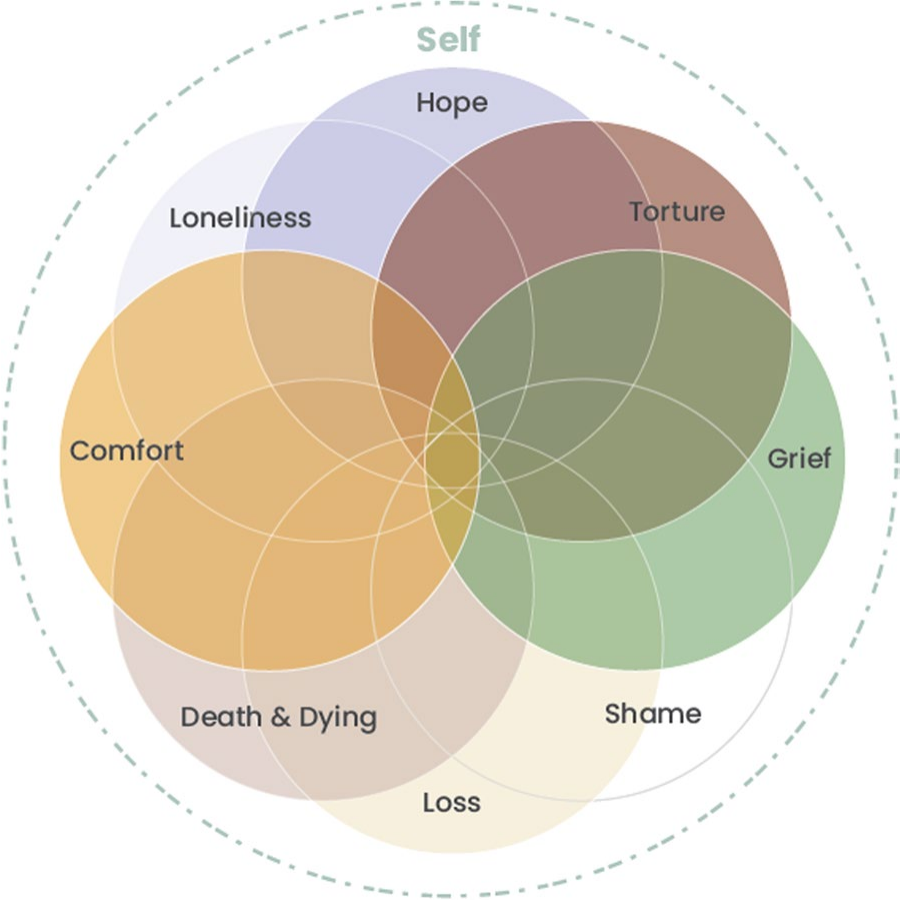
Complex Kaleidoscope of artist’s AN experience

Using the artist’s medium of colour, thematic representations identified within the paintings as a result of the analysis were identified and analyzed, using Photoshop [79]. A digital ‘RGB’ colour code was denoted to each theme, generating a colour map. For example, the participant described themselves as *‘I am blue. Blue, like a greeny blue’* and a representative colour sample was extracted from her selected image to represent ‘*self* *as colour’* (Image 15). This communication of findings is in keeping with an ‘experience near’ [80] representation of the SE-AN phenomena. The SE-AN Kaleidoscope (see Fig. 1), affords a visual display of the themes in this analysis, using the artist’s chosen colour-wheel, influencing the lens through which they experienced the world. The artist communicated via metaphor and colour, and so the Kaleidoscope metaphor honours this by depicting the multi-dimensional, shifting tensions of AN, through the lens of the artist’s severe and enduring experience.

### Findings

**Fig. 2:**
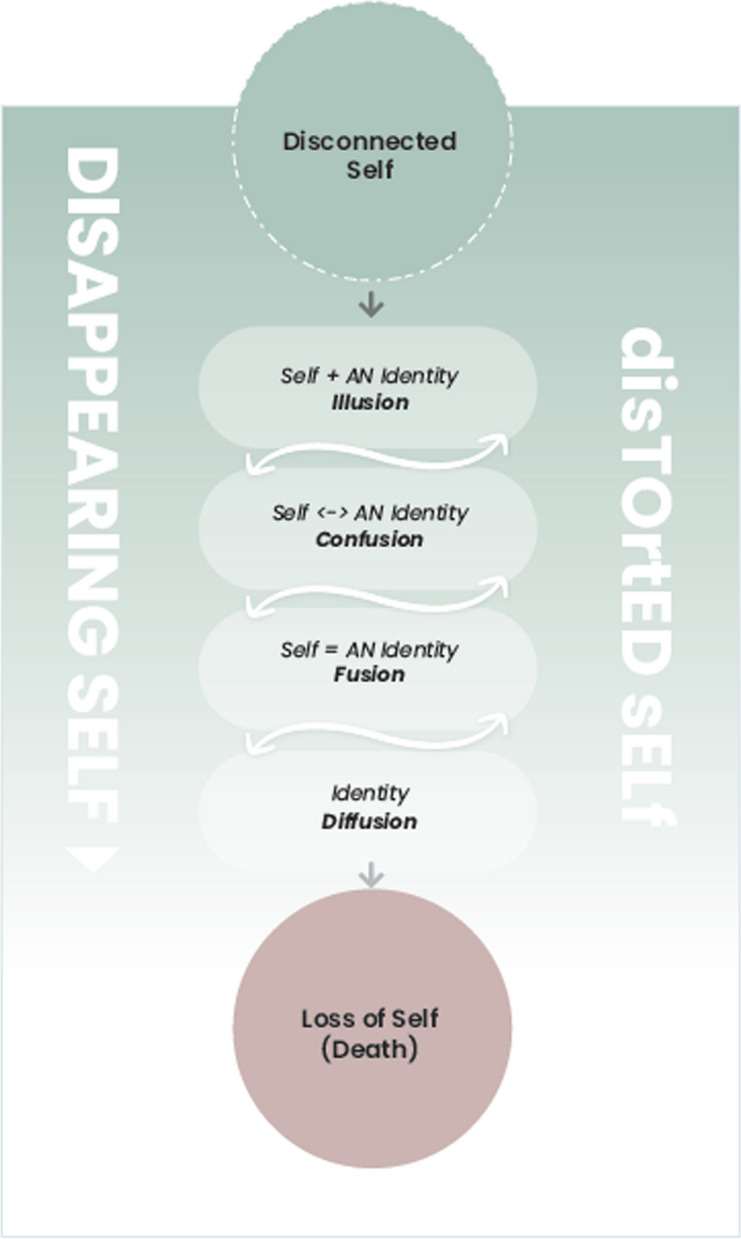
Self and identity processes in SE-AN: illusion, confusion, fusion, diffusion

The process of analysis induced three superordinate themes, with two sub-themes within theme 1 (Table 2). Two cross-cutting themes (A & B) traversed the findings, whereby the cross-cutting theme B - ‘colour’ generated the previously described SE-AN Kaleidoscope (Fig. 1). Collectively all themes contributed to the development of a graphical conceptualization of self-disturbance in SE-AN, as interpreted from the artist’s work (Fig. 2 – self and identity processes in SE-AN: illusion, confusion, fusion, diffusion).

**Table 2:** Themes in SE-AN

Cross-cutting theme A	Themes and sub-themes		Cross-cutting theme B
Shifting hope	1	Disappearing self: ‘lost in a big world’	Colour
	*1a*	*Embodied intra-psychic processes: vulnerable self*	
	*1b*	*Grief: loss of self*	
	2	Dialectical dilemma	
	3	Death and dying: finding meaning

#### Theme 1 ‘Disappearing’ self: ‘lost in a big world’

**Image 1:**
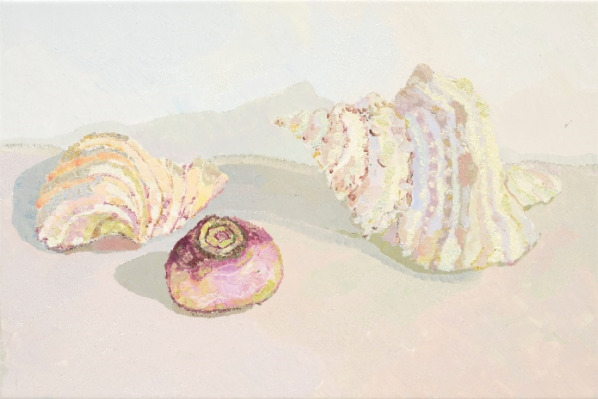
Shell, turnip, and another shell, 2013, oil on tile, 30 × 45 cm

*‘I am a shell…A creature inside the shell. A little, little thing, lost in a big world’.* This expression, connected to Image 1, indicates an ‘*identity-self confusion*’ (see Fig. 2). Depicted is an uncertainty as to whether the artist was the shell (chosen AN metaphor) or the creature (‘self’), within. Referring to herself as a ‘little’, non-specific ‘thing’, illustrative of her grappling with a sense of herself and her value as a person. Also communicated was a sense of the artist’s phenomenological experience of feeling ‘lost in a big world’ indicating a loss of self, to a world for which the vastness was experienced as overwhelming to her.

**Image 2:**
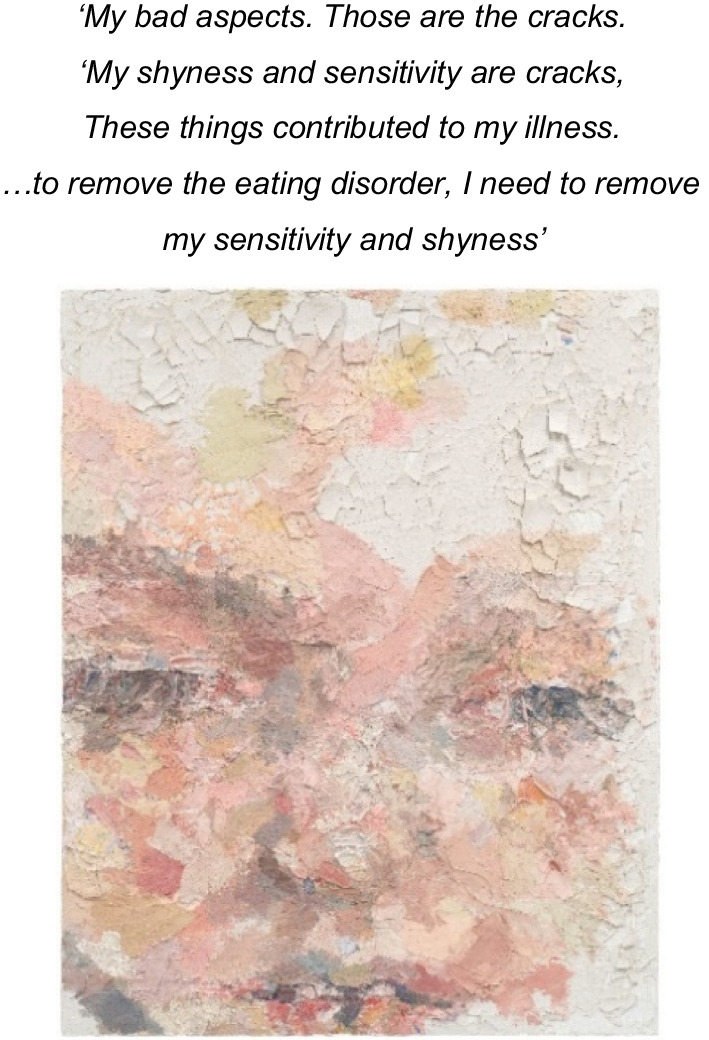
Untitled, 2008, oil on clay, 76 × 61 cm. Note this clay image is impermanent, designed to crack and disintegrate

An exploratory self-portrait, (Image 2), communicated a sense of fragmentation of the self by the pervasive AN identity through words and imagery. This poignantly named self-portrait, ‘Untitled’ (Image 2), is impermanent. The canvas is overlayed with clay, engineered by the artist to crack and disintegrate. The self is portrayed as fragile and evanescent. Also communicated is an ‘*identity self-fusion*’ with AN (see Fig. 2). Perceiving that loss of AN required elimination of attributes perceived as intrinsic to the self, ‘*sensitivity and shyness*’, such that without AN she would disappear; ‘*The eating disorder has taken over me completely now. It’s taken over me’.* This kinetic work further abstracts the faintly distinguishable face. The calcified clay remains erase the image over time, towards a ‘loss of face’.

**Image 3:**
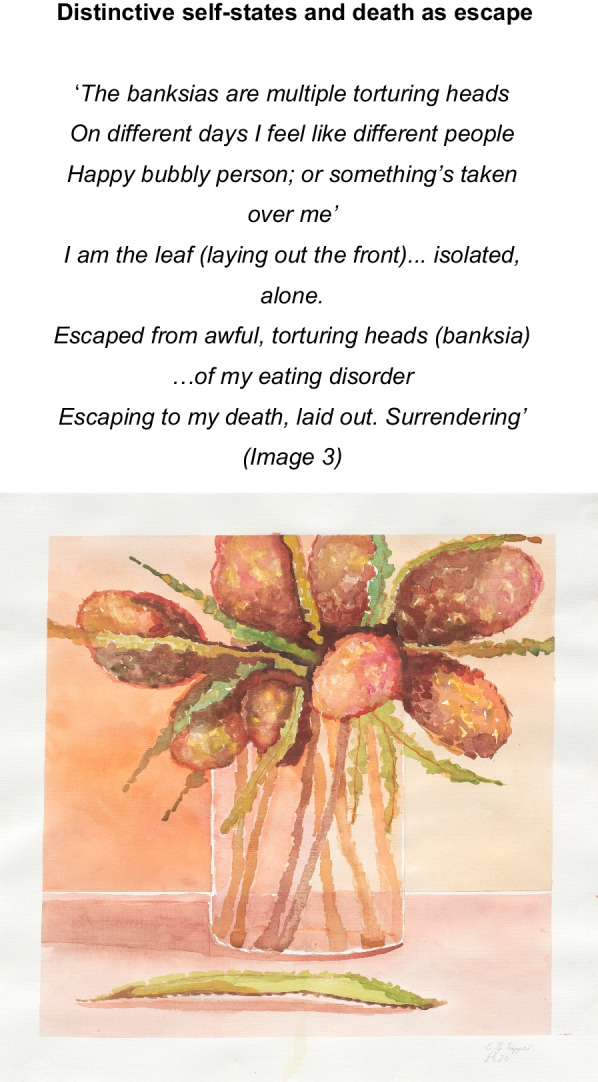
Banksia Bunch, 2020, Watercolour on paper 39 × 39 cm

**Image 4:**
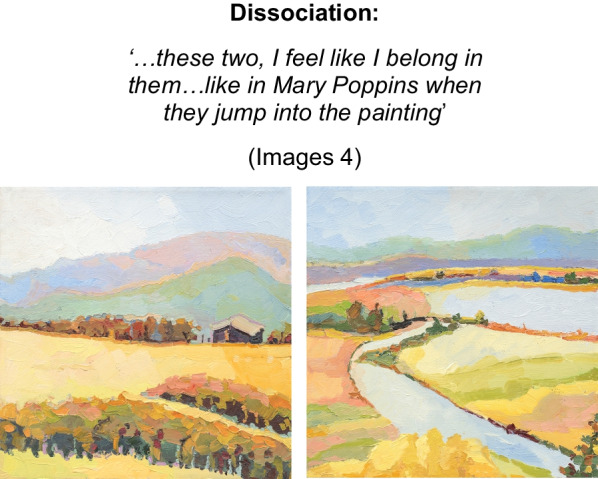
Landscape 1 and 2, 2013, oil on canvas, 30 × 30 cm

**Image 5:**
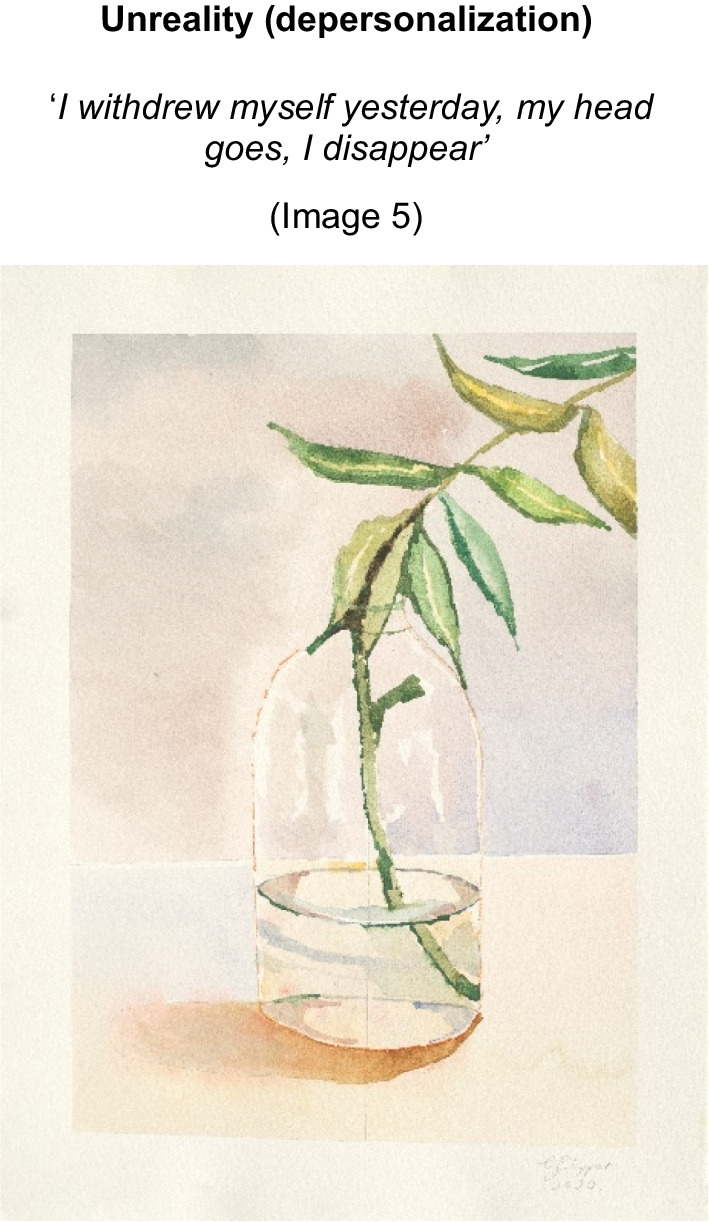
A lone leaf in vase, 2020, 39 × 29 cm

The artist sought to ‘*escape that awful feeling, torturing thoughts… of my eating disorder*’ (Image 3) via disconnection from mind and body. Inter-related processes of dissociation (Image 4) into distinctive self-states i.e., ‘*multiple torturing heads…on different days I feel like different people*’ (image 3), unreality (de-personalization) (image 5), and death as escape (image 3) are manifest through the artists words and paintings. We have termed this state *identity-self diffusion* (See Fig. 2), to describe this recursive process of fragmentation of self.

**Image 6:**
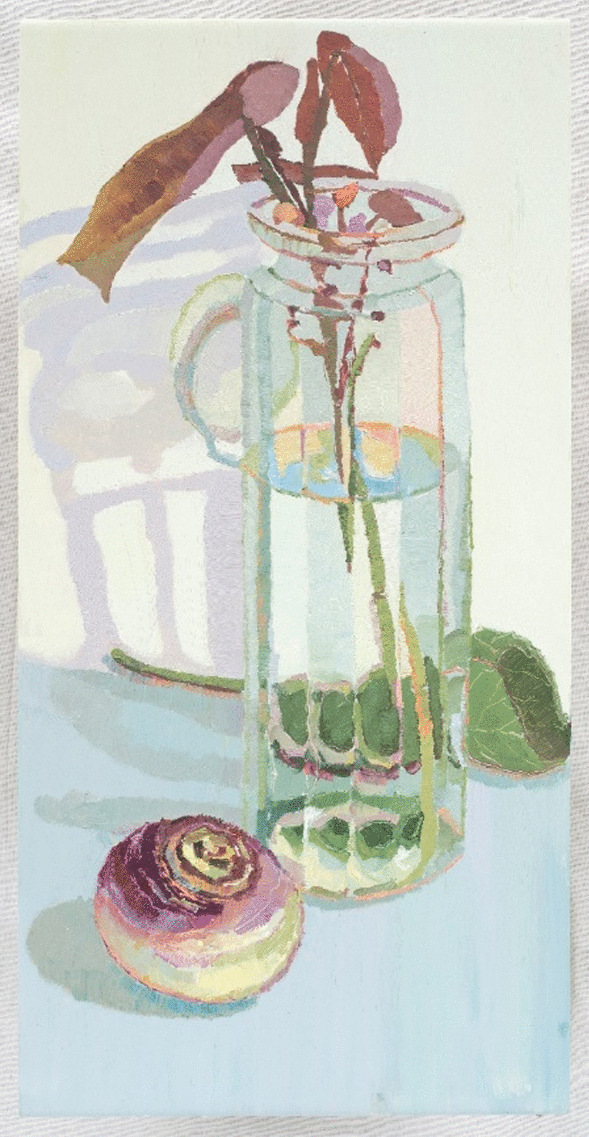
Leaf in spaghetti jar, 2013, oil on tile, 45 × 30 cm

The artist acknowledged seeing the self ‘*through the distorted lens of the vase’*, perceiving that *‘I am not kind or caring, I do bad things’*, (to protect my AN identity). She was aware of her altered self-perception, yet appears impervious to alternate self-views conveyed to her, for example sharing that ‘*mum says stop being so sensitive*’. In reference to Image 6, the artist acknowledged that *‘I can’t see clearly …’* and remarked ‘*… I have distortions…things appear distorted and more interesting’*. There is a sense of valuing the distorted AN perception, rendering the self as more interesting. The image is dimensionally long and thin, compared to her other works. It is heavy yet fragile, painted on a ceramic tile. These qualities exemplify the paradoxes and deception within the artist’s expressed distortions.

**Image 7:**
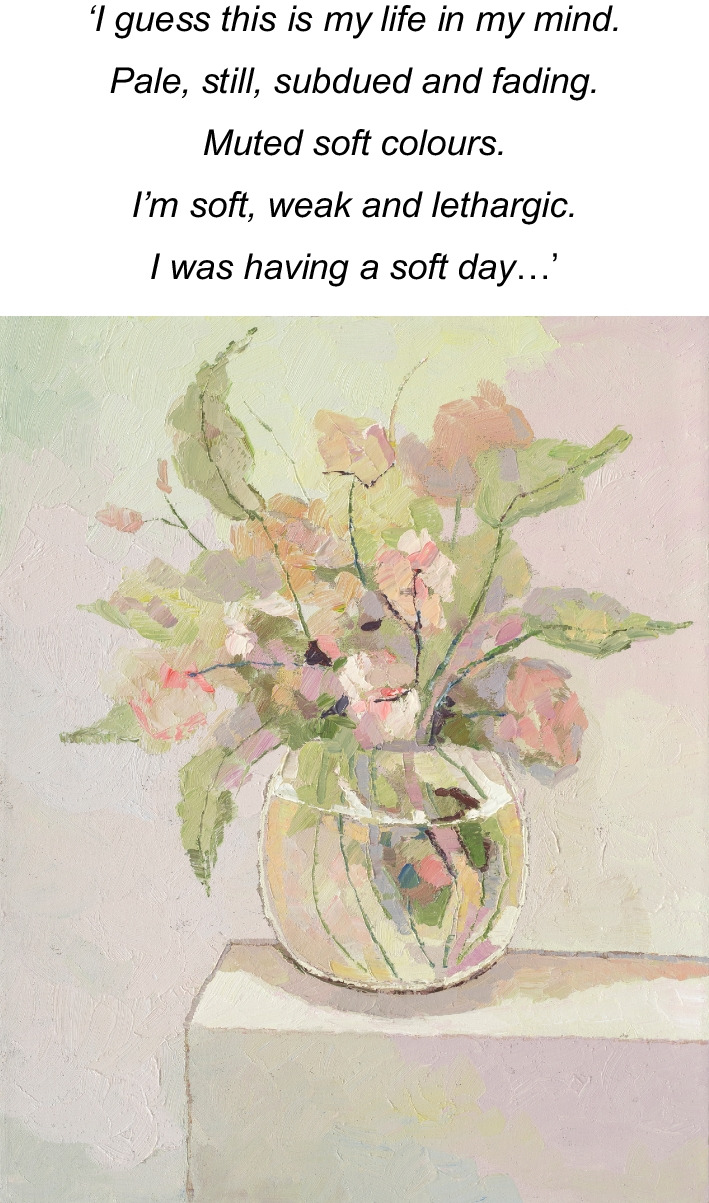
Still life with flowers, 2020, oil on linen, 50 × 40 cm

**Image 8:**
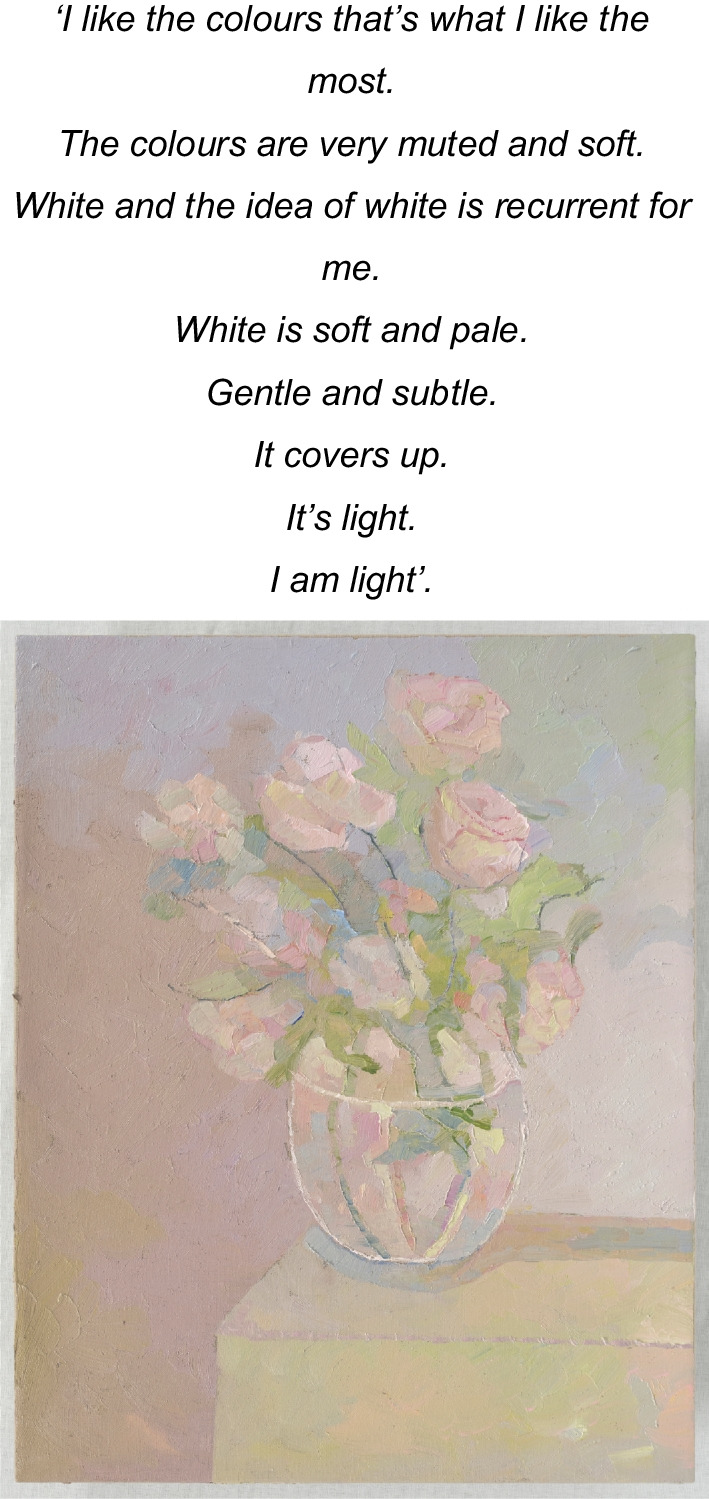
Pale still life with flowers, 2020, oil on linen, 50 × 40 cm

The artist’s final ‘twin’ series, (Image 7 & 8), painted the mark of her end of life. The processes of acceptance of self as dominated by AN (still life with flowers) in contrast to seeing or reconciling ‘self’ through her artworks (pale still life with flowers) depicted a tension within herself. She projected her ‘disowned’ inner beauty (true self) onto the canvas within these images, which are unequivocally beautiful in their feminine elegance. Yet, she is unable to see or accept the self as beautiful in her lived reality, distorted by the AN lens. This skewed self-view represents a self-distortion, beyond body image and is considered consequential to the AN identity-self fusion.

These paintings (7 and 8) with the artist’s emphasis on *‘muted’* and *‘soft’* colours and the recurrent theme of ‘*white and the idea of white*’ depict the self as ‘*soft’, ‘pale’, ‘gentle’, ‘subtle’, ‘light’* and yet also ‘*cover(ed) up*’ by these colours. The artist stated, *‘I actually like it’* and in the final sentence she concluded with ‘*I am light*’, in contrast to ‘*heavy’* signifying that AN was embodied as an idealized self in the final stages of the disappearing self as lived in this world. Through her words and paintings, the artist depicted this process of a disappearing self through (a) embodied intrapsychic processes; and (b) grief of a loss of the self to the AN. Thus, the self progressively disappeared, disturbed by the SE-AN experience (See Fig. 2).

#### Sub-theme 1a: embodied intra-psychic processes

**Image 9:**
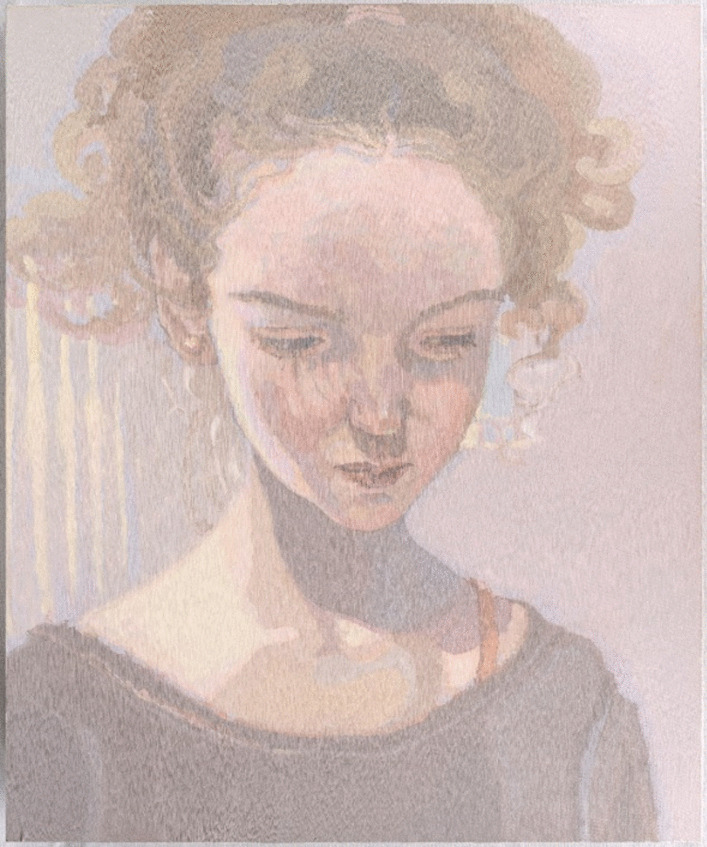
Self-portrait large, 2009, oil on canvas with silk and hair, 120 × 100 cm

*‘It puts a veil over me. To hide something. To hide myself… to mute my colours’* (Image 9).

The artist’s words and paintings signify internal feelings of distress via shame processes, sensitivity and negative self-evaluation, which were embodied. Shame, [81], is described as acutely disturbing to the self and a silencing, isolating emotion. Applying this definition, the presence of shame was implicit in the artist’s narrative as exemplified in the quote above. She articulated wanting to ‘*disappear’, ‘cover up’* and *‘hide’* expressing discomfort in a physical existence, hesitant to *‘take up… space’* even through her artworks (Image 9). This painting, titled ‘self-portrait large’, is three times her usual scale. Extending upon the notion of a distortion in body image to a more global distortion in her self-image.

The underlying shame processes were also recognized in a hesitance to exist and unworthiness; ‘*I don’t want to disturb anything or anyone’* (image 1). The artist’s perceived, fundamental inadequacy (shamed self) and vulnerability were hidden via AN. This offered her *a ‘protection from the world’.* The unaddressed shame thus became alienating ‘*I don’t want people to see that side of me’,* defeating ‘*I can’t reach out and be with others’* and undermined a sense of belonging ‘*I am a still life…. I stay mostly in my home’.*

‘Sensitivity’ was articulated verbally by the artist and is undeniable in the thoughtfully considered rendering of her artworks. She dichotomously stated; *‘my bad aspects…my shyness and sensitivity’’,* judging her sensitivity to be ‘*bad*’ and to be rid of ‘*I need to remove my sensitivity and shyness’ (see image 2*). Thus, change became dominated by changing aspects of the self that were perceived as unacceptable to the self. Furthermore, the artist approached herself with self judgement, with a rejection of parts of the self (‘*this part of me is not OK’*) and a fixed-negative self-view: ‘‘*I am not kind or caring. I do bad things. I can’t get better*’. The struggle to recover appeared to contribute to this negative self-view that recursively obscured recovery as a possibility. Implicit in this struggle was reduced self-compassion and an impermeability to alternative self-views*.*

Reminiscing on a self-portrait (image 9), painted when ‘*I was well… my face full… between hospital stays’*, the artist remarked that despite being physically weight-restored she remained ‘*sad, pale, subdued, washed out, faded’.* Later stating ‘*it’s not helpful to force-feed’;* taking a position on the treatment that sought to cure but inadvertently harmed her. Ultimately, resigning herself to the impossibility of ‘normal’, describing that ‘*the distance is never-ending between me and the hill. It’s impossible’* (image 3). Thus, she lost sight of what and where recovery was for herself. Notably this is the only painting the artist explicitly related to AN treatment.

As her hopes to be ‘normal’ diminished, her relationship with hope shifted [77] towards hope to unburden herself from this world towards lightness (‘*I am light’*; Theme 1, Image 8) and to leave a legacy through her book for those with AN. Her final words in the book offered wisdom of a life with AN:

*‘I was unwell for a while before I got treatment.*

*its not helpful to force-feed…*

*Try to open up a bit and get some help.*

*I had nowhere to take my worries. I felt lonely. I kept it all inside*

*Find people that are nice and treat you well’*

The artist offered a subtle call to transform treatments, stating that forced feeding was experienced as unhelpful. She implied that she wasn’t aways ‘treated well’ in treatment, but offering hope that there are people who treat you well and to keep looking.

**Image 10:**
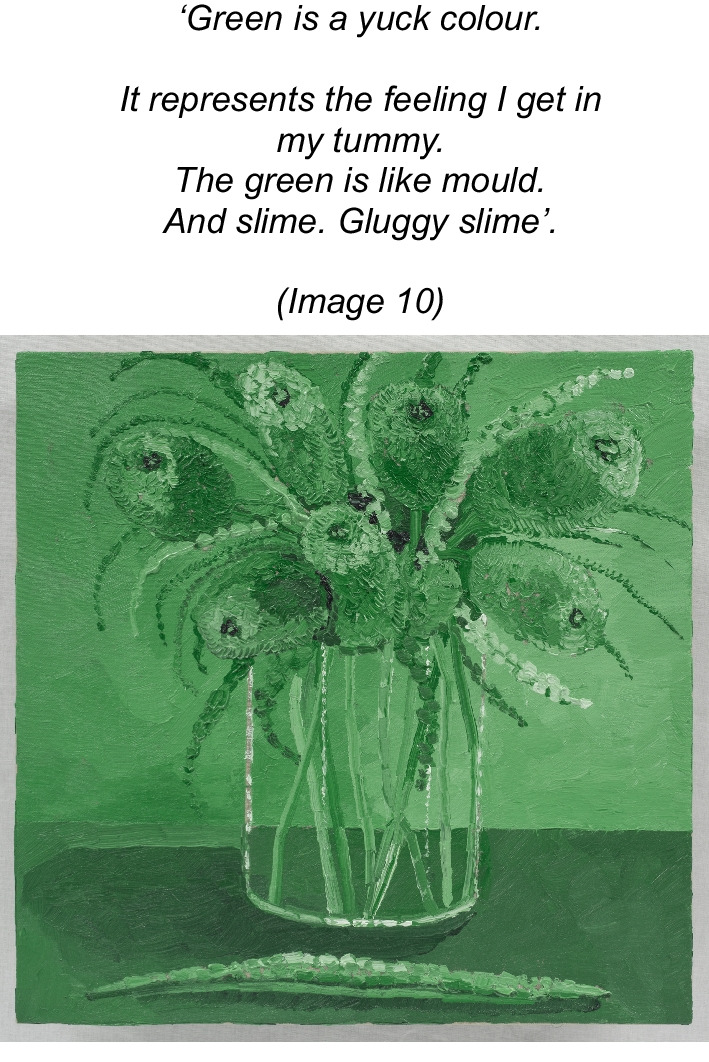
Banksia Monochrome, 2016, oil on linen, 40 × 40 cm

The artist described her feelings as represented through colour (Image 10), selecting a colour she disliked and held in the metaphors of ‘*mould’* and *‘gluggy slime’.* She located the aversive feeling to her stomach, giving the impression the feelings are old and pervasive.

In this narrative, it appeared that the artist identified and expressed her feelings yet could not accept them. Her feelings were something to be rid of ‘*the feelings got out of me’* and experienced as aversive, pervasive, non-finite and somaticized.

#### Sub-theme 1b grief: loss of self

**Image 11:**
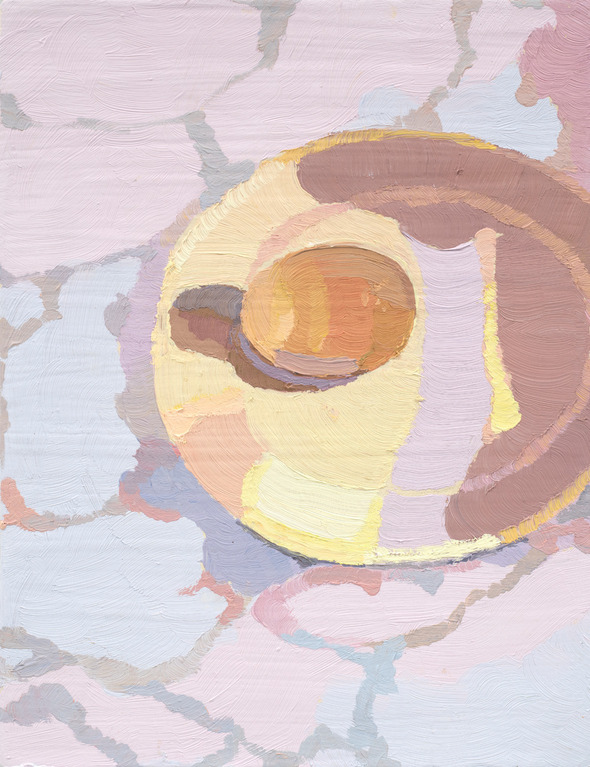
White on white, egg on plate, 2009, oil on gesso board, 26 × 20 cm

The artist’s account of a life lived with prolonged AN was pervaded by grief of a loss of self and identity. Here, the artwork is a paradoxical embodiment—her depiction of the grief of her loss of self and her efforts to resist this loss of self. She attempted to hold onto herself, even if this was through her fragments of lost hair ‘*I have a jar of my own hair… I have embroidered it into some of my paintings* (Image 9). The artist described cumulative and non-finite losses of herself and her identity to anorexia nervosa, where she experienced a loss of her ‘assumptive world’ through ‘*loss of getting fertile’ (image 11).*

As depicted throughout the narrative e.g. in images 1,2,8 9 and 10, the artist grapples with a loss of self, hesitant to exist and ultimately lost to the anorexia;

‘I am empty…

…Lost in a big world… (Image 1)

The eating disorder has taken over completely now,

It’s taken over me’ (Image 2)

#### Theme 2: dialectical dilemma

**Image 12:**
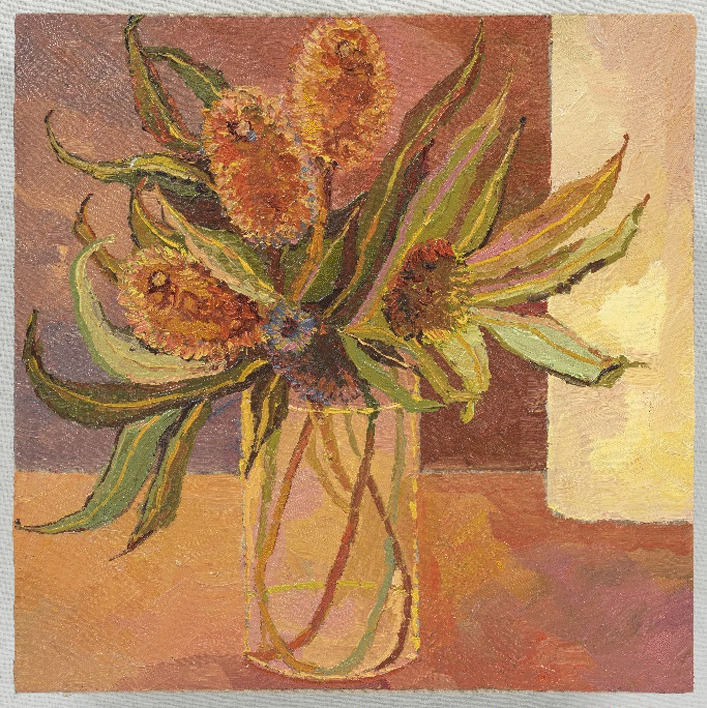
Banksia 1, 2020, oil on linen, 40 × 40 cm

The artist drew on representation through colour and the metaphors of picked flowers and shells, to articulate a ‘dialectical dilemma’. Two polarized truths existed for the artist, forming the dialectic. AN is perceived as *‘safe’ ‘protective’* ‘*familiar’, ‘homely’ ‘warm’* ‘*comforting’, ‘cuddling’* captured within image 11 and in parallel, AN is *‘heavy’* ‘*fragile’* ‘*prickly’, ‘torturing’ and ‘limit(ing)’ (See images 1, 4, 9 and 12).*

**Image 13:**
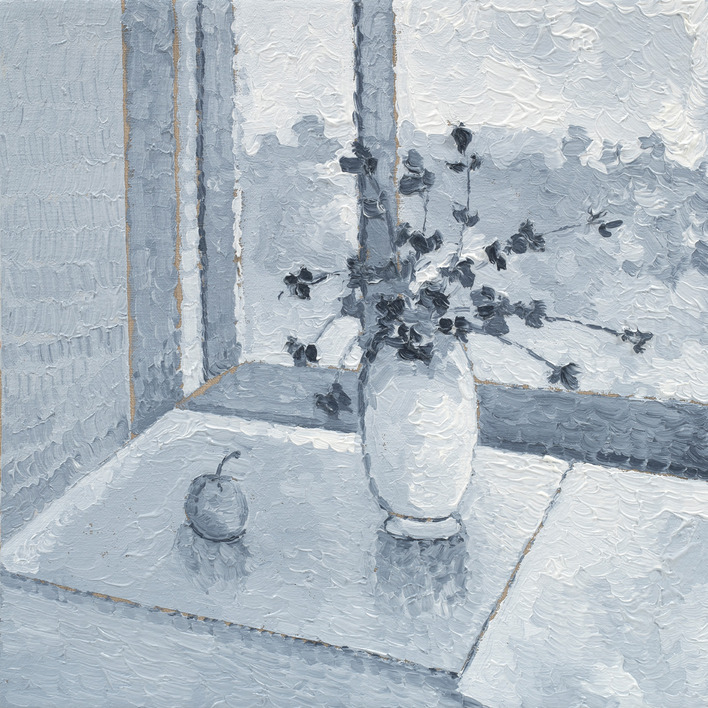
Vase in front of window, 2019, oil on canvas, 30 × 26 cm

Thus, rendering the artist who is ‘*sensitive, soft, gentle, fragile, light and subtle’* (see image 7 and 8) to feel ‘*isolated…lonely’ ‘sad’, ‘withdrawn’, ‘muted’*, *‘empty’, ‘still’, ‘taken over’, ‘subdued’,* ‘*faded*’ *and ‘pale’ (Images 3, 8, 11 and 13)’.* Ultimately re-enforcing the AN through perpetuating a lost sense of herself and loss of hope ‘*normal was a long time ago’* (Image 10). The self, described and depicted as ‘*disappearing’ (*theme 1*).*

**Image 14:**
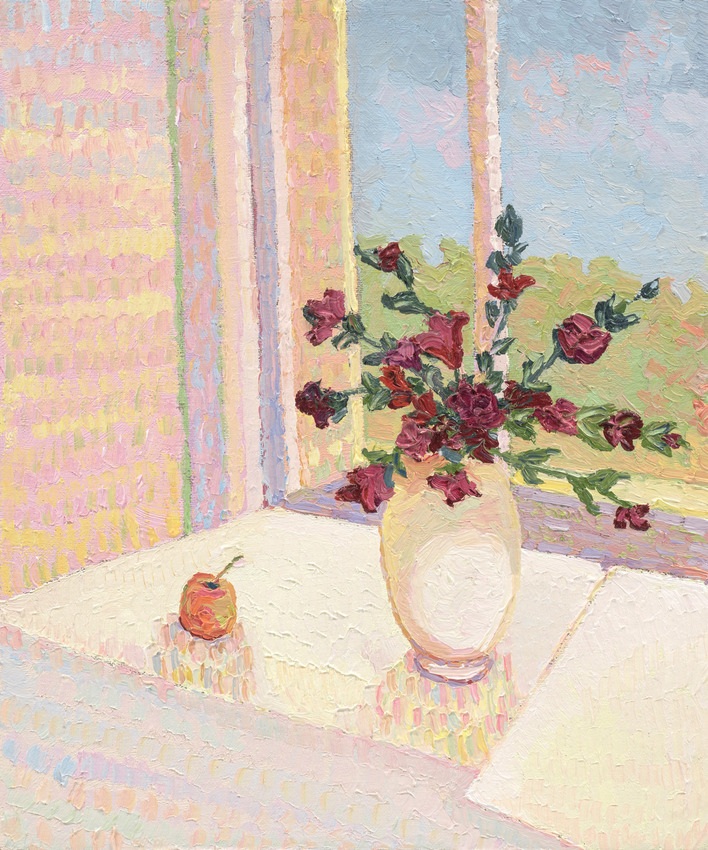
Looking out my studio window, 2019, oil on canvas, 30 × 26 cm

Within the dialectical conflict, the artist described an impasse; *‘frozen in time’, ‘I am a still life’, ‘I am stuck’.* Again, twin images (13 and 14) with and without colour, articulate this dual reality, reconciling a ‘*safe*’ yet limited life ‘*I stay mostly in my home*’ with ‘*feel(ing) content’*.

The artist described being *‘milked’* of nourishment, via the metaphor of colorlessness (black and white) and elsewhere, fading colours (e.g., images 7 and 8) communicated the dialectic. Representative of loss of engagement in life and that which sustains life—food and connection with others. She holds onto a painted world, adding depth and colour to life ‘*as though sitting on a cake… thick layers of paint…painting makes me feel better…so I do it’.* Painting offered a portal to a curated, enriched and safe world, within the artist’s control.

#### Theme 3: death and dying: finding meaning

**Image 15:**
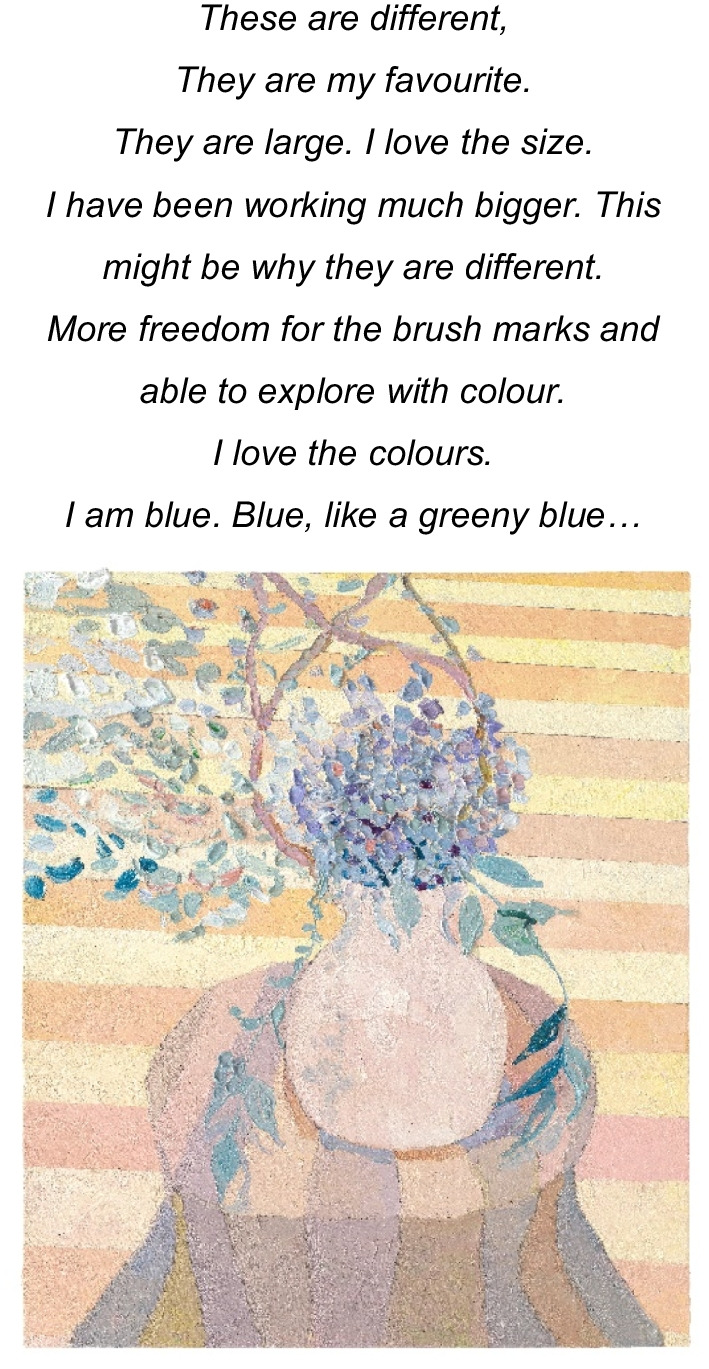
Untitled 3, 2020, oil on linen, 70 × 60

Death and dying was introduced through colour; *‘The colours are dead colours’* (See image 3). Within this exploration of death, the artist acknowledged that little of them remains and that inevitably they will wither and die; ‘*You pick flowers… they dry out and decompose.’* The flowers are ‘picked’ and therefore displaced from that which nourishes them, hastening their death. In the late stages of the narrative, the artist connected with a deceased artist, Margaret Preston (29 April 1875–28 May 1963), drawing inspiration from her body of work [82] ‘… *I’ve been reading her everyday… I was inspired’.* Within a knowing that death is imminent ‘*sometimes I wake up and I think oh my gosh I’m still here’,* the artist sought connection to a representation of death (MP) and made her mark, finding meaning via the creation of her legacy ‘*a bit of a biography…. They have quotes by her…. I’d like to do that in [my] book’.* Her penultimate artworks became bigger, bolder, colourful and the artist spoke more generously of her own work and finding a sense of self through colour’ ‘I am blue’ and ‘*freedom*’ through ‘*the brush marks’* and being able to ‘*explore with colour’* (Image 15).

### Cross-cutting themes

#### Shifts in hope

**Fig. 3:**
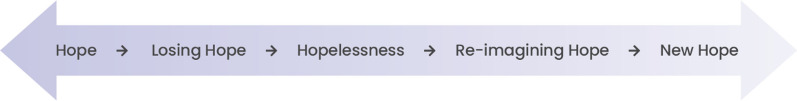
Artists shifts in hope

The artist expressed an enduring desire for things to be different; *‘I would like to shine, but I can’t’ (Image 1)* and *‘I wish it (normal) was closer’* (image 3). This was held alongside the diminishing hope of a ‘*normal*’ life she once knew ‘*a long time ago*’ (image 10)*.* The artist authored a ‘narrative identity’ [83] through her ‘living metaphor’ as a comforting, internalized life story which can offer a sense of unity, purpose and meaning making, in the face of her suffering. Within her autobiographical legacy the artist reclaims the disappearing embodied self through art. The corporeal self is lost but an interpretivist self is created and immortalized through painting. Thus, offering a ‘new hope’—to herself and to others. Hope is reallocated (White, 2007) from the desire for her own ‘recovery’ to the hope of leaving a legacy for those with a lived experience and those who care for and treat them (see Fig. 3).

## Discussion

This study offers a unique and rich insight into a life lived with SE-AN. The artist created a ‘living metaphor’ and ‘narrative identity’ [84] through her artworks which unveiled her innermost experience. She then interpreted this with spoken language to communicate her experience with others which enabled interpretation via a phenomenological analysis, to reveal a theoretical understanding of the SE-AN experience.

A central theme within this work was the phenomena of a ‘disappearing self’, interacting with embodied intra-psychic processes, and the dialectical dilemma, toward the loss of self. SE-AN is conceptualised as ‘self-organising’, but the self-substituting AN furthers the disconnection from the self, including dissociative processes, and is thus re-enforcing (See Fig. 2). The hesitant self progressively disappears, trapped in the knowingness of a dialectical dilemma. Here, the illness is perceived as ‘*safe*’ yet ‘*torturou*s’, creating an impasse. This left the artist ‘*empty*’—a ‘*shell’* of themselves—through cumulative losses, including a loss of hope. This culminates in a loss of self to the AN identity see Fig. 2 (self and identity disturbance in SE-AN). Alongside the loss of self, the artist re-imagines hope (See Fig. 3), creating a new hope, by finding meaning in their life lived with AN through a legacy that can be shared with others and messages of hope for recovery. Colour and metaphor paint the picture, further illuminating the artist's phenomenological constructs. The SE-AN Kaleidoscope (Fig. 1) shows the unique interaction of the aspects of AN portrayed by the artist, illustrating the illness complexity as seen from the perspective of a lived experience, imploring sensitivity to these processes in treatment.

### Discussion of themes

The ‘disappearing self’ houses the sub-theme embodied intra-psychic process: vulnerable sense of self, that build a picture of ‘self-disturbance’. Here, the artist struggles to be in her mind and body and find a place in the world. This extends upon existing understandings of genetic, neuro-bio-psycho-social models of AN [85], as applied to SE-AN. Underexplored causal and maintaining factors highlighted in this paper may include complex intra-psychic processes for example shame [86], and interactions of a ‘global sensitivity’ with embodied processes of interoception, alexithymia, somatization and dissociation. SE-AN being a chronic illness is confounded by loss and grief to the illness experience, which furthers the disturbances in self and identity. The impact of chronic illness on identity disturbance has been explored outside the field of eating disorders [87]. Thus, this study finds that in SE-AN ‘self-disturbance’, as part of a ‘global impoverishment’, are additional putative factors of severity, influenced by protracted duration.

The intrapsychic process of shame has been explored in ED literature theoretically [86], phenomenologically [88–90], and empirically [91]. A recent meta-analysis [92] and systematic reviews of shame [93, 94] support the importance of shame processes in eating disorders, including AN. Bryant and colleagues [56] in a phenomenological AN synthesis, inclusive of children and adults with AN of unspecified duration, also found themes of shame (both internalised and body-related), as phenomena in AN. The present study and our synthesis of SE-AN experiences [17] identifies shame as a persistent feature in SE-AN.

By virtue of being an alienating experience [81], shame can thwart treatment engagement and is cited as such [19, 95]. This may account, in part, for low uptake of treatments (e.g. 50% [96]) and high drop out. Additionally, the expression of shame is of itself shameful and therefore challenging to resolve. Communication via visual means appeared to offer an alternative avenue of expression for the artist, consistent with syntheses of art therapy for EDs [97, 98]. The use of visual art therapy is understudied and has potential to bypass intellectualisation and cognitive filtering, enabling unconscious representations to emerge [99] and potentially re-processed, which may include shame processes.

Sensitivity was a thread throughout the artist’s explorations and has been identified more broadly as a vulnerability trait in AN [51, 100]. Less is known about this intrapsychic process and its role in the progression to SE-AN, however it is implicated in increased severity [100]. Sensory Processing Sensitivity (SPS) is a term to encompass all facets of sensitivity—stimuli, interpersonal, sensory, affective and perceptual. It is considered a heritable trait, more pronounced in females and can be measured using the validated, Highly Sensitive Person Scale (HSPS)[101]. Interpersonal sensitivity in AN recognises heightened acuity to rejection and punishment [102], whereas sensory sensitivity is an elevated subjective awareness of somatosensory stimuli [103]. Affective sensitivity is where feelings are experienced more deeply or to extremes and perceptual sensitivity, being the ability to detect negligible or low intensity stimuli in the environment and process these to a greater depth. These may additionally interact with empathic processes [104, 105]. The presence of heightened sensitivity is at odds with the notion of poor interoception and alexithymia in AN, [48] which may account for inconclusive findings regarding alexithymia, when controlled for mood and acute starvation in AN [106–108]. As an alternative hypothesis and as presented in this study, the person with AN may feel and perceive more deeply (highly sensitive) and thus, emotion is experienced more globally, intensely, and somatically, overwhelming a person and therefore more difficult to process and put into words. Adaptations to withstand SPS may emerge by necessity to support the sensitive person in the world. It is well established that AN can dampen stimuli [109–111] representing a favourable and useful ‘functional adaptation’ [17], p. 11 of the starved state, where sensitivities or intolerances to somatic states may otherwise be heightened [103, 112]. This perspective is supported by Zucker et al. [50], who found that irrespective of BMI, those with AN reported greater sensitivity to sensation and were more likely to actively avoid stimuli than the people in the healthy control group. Purging behaviours may also represent an adaptation to managing this visceral, somatisized emotional experience and studies have shown altered visceral interoception in AN, indicating further study [112]. Thus, ‘high sensitivity’ may intensify the somatic experience and subsequent overwhelm may necessitate a disconnection from the self (dissociation) and avoidance in interpersonal relationships (Images 3,4 &5). How these constructs interact with neurobiological findings [113–115] and translation to treatment is incomplete [116–118].

Skaargund (2007) [119] in a phenomenological series of AN, introduced the term ‘concretised emotions’ to describe how emotional (psychic) and physical phenomena are fused as one. Emotional responses may be interpreted via a fixed framework, disallowing spontaneous experiences. In the present study, the artist describes an embodied sensation (in their stomach) and ascribes an aversive, visceral response to this sensation ‘*yuck, glug, slime’*, that is to be rid of ‘*the feelings got out of me’*. Hence, there is also a need to distinguish between the ambiguity of interoceptive experiences versus non-acceptance of them as related to AN [120]. An alternative perspective to ‘poor interoception’ and alexithymia is supported by the findings in the present study (Image 10), where feelings were ‘stuck’ and somaticized.

Dissociation, also a form of disconnection, is additionally underexplored in AN, despite research estimating prevalence up to 29% [121]. Psycho-form dissociation (See Additional file 1) is linked to poor treatment outcomes [121], implicating SE-AN. In the present study, feelings were somaticized (Image 10) and recent research (e.g. Longo and Colleagues [122]) has explored somatoform dissociation, given somatic aspects inherent to AN [122, 123]. Longo et al. utilized a severe and enduring group of people, distinguished via mean illness duration of 6 years and at least two prior treatments. Based on results, careful assessment of both psycho-form and somatoform dissociation in AN is recommended, implying dissociation-informed interventions in care. Findings by Meneguzzo et al. [124] demonstrated improvements in dissociation symptoms to good effect sizes (further elevated in patients with higher alexithymia scores), when treatments (e.g. CBT-E) were adapted to included mindfulness approaches. They recommend assessment and specialised treatment of these phenomena. These findings also affirm that top-down cognitive behavioural treatment models may serve to reinforce intellectualisation, resulting in further disconnection from a felt sense of self [125] and self-expression.

A conceptualisation of the interruptions to self and identity as part of the SE-AN experience is introduced in Fig. 2. Evident in the artist’s work, is the progression of AN from being a pseudo/substitute identity or ‘identity illusion’ with the emergence of ‘identity confusion’ that progressed to totalisation of the self in ‘identity fusion’. Hence duration of the AN, is implicated in perpetuating and maintaining the AN. Various stages of progression to SE-AN were characterised by self-processes. Dissociation (fragmentation, de-personalisation, un-reality, and distinct, polarised states of being) influenced the diffusion of identity and loss of self (see Fig. 2). This represents an intensifying of the self-fragmentation, as AN advanced.

Addressing identity has been the focus of the later phases of AN treatments and following weight restoration for adolescent and adult AN (e.g. Family-Based Therapy (FBT) for adolescent AN—[126] & the Maudsley Model for Anorexia Nervosa Treatment for Adults (MANTRA [42]). It is a long held clinical belief that weight restoration is required before other aspects of the AN experience can be addressed therapeutically. Typically, people with a BMI < 15kgm2 are often not included in outpatient treatment or treatment trials [24, 127, 128], irrespective of weight stability. However, in the present study, the artist, who communicated profound insights, including the paintings and words in her book at a BMI < 12 kg/m2, which was maintained for more than 5 years. This provides evidence that some people may adjust both physically and cognitively to being underweight. This is a finding also consistent with our SE-AN synthesis [17], where participants needed to first build a trusting therapeutic relationship (which took considerable time), that could withstand ruptures, and also understand themselves within the (dys)functional role AN has in their life, in order to relinquish it. Failure to build insight in parallel to weight restoration was an aspect described as treatment ineffectiveness by participants, influencing relapse and loss of hope [11]. This delicate balance has long been recognised [129], however consideration to strengthening an identity outside of the anorexia nervosa identity earlier in treatments, has not translated. Whether this may facilitate sustained improvements of ED symptoms is a hypothesis that remains to be tested. A previous randomised control trial [130] targeting identity development, utilised a cognitive behavioural model for development of ‘self-schemas’ by way of improving emotional health in women with AN. This was found to decrease desire for thinness with subsequent increases in psychological well-being. Subsequently, a novel treatment intervention, Specialist Psychotherapy with Emotion Kent and Sussex (SPEAKS) [127] is in pilot. The treatment is centred upon strengthening the person’s emotional self [55] and combines integrative emotion focused therapy and dialogically based, schema ‘self-parts’ therapy. A pilot evaluation of narrative therapy, which has not been extensively researched [131] is also underway [132]). A key tenet of narrative therapy is addressing a person’s identity development through re-authoring conversations to develop and thicken stories obscured by the dominant problem story [77]. Results of these trials may be a first step toward understanding the role of these neglected aspects of treatment.

### Clinical implications: SE-AN diagnostic

Currently SE-AN is conceptualised on a continuum of AN, with severity, duration, and treatment exposure specifiers. Based on the findings of this study, our synthesis of studies [17] and LE perspectives of distinguishing features in labelling and defining SE-AN [133], we propose that the conceptualisation of SE-AN is incomplete. Additional criteria are needed to capture (i) the self-disturbance as acknowledged beyond body image disturbance (ii) the self as subsumed to the disorder as part of the *global impoverishment* of self, across life domains to the AN.

The introduction of these aspects could modify the SE-AN diagnostic, via a refinement of the severity criteria. We would suggest that irrespective of BMI, a duration > 3 years of AN (i.e. persistent state of dietary restriction’ and or compensatory behaviours) can sufficiently elicit sequalae of a ‘*global impoverishment’*, including (a) significant impairment to physical, personal, family, social, educational, occupational, fiscal or other important areas of functioning, including capacity to live independently and (b) Marked discontinuity in sense of self and agency, where ‘self’ is subsumed to the disorder. In SE-AN, we purport a progression from the corporeal ‘*disturbance in the way in which one’s body weight or shape is experienced *[11]* (*AN) and ‘*overvaluation of weight/shape’*[9]*,* to a more global, embodied disturbance of ‘self’. Whereby a profound ambivalence and fear manifests in relinquishing the AN, as it becomes intrinsic to a person’s sense of self and perceived phenomenologically as a total ‘loss of self’.

The present study is an example of a person who engaged in all facets of available treatment throughout the lifespan of their AN and who did not experience this as curative. As such, further caveats are needed regarding treatment exposure specifiers in the SE-AN diagnostic, and recovery parameters. As it currently stands diagnostically, ‘*exposure to at least two evidence-based treatments appropriately delivered together with a diagnostic assessment and formulation that incorporates an assessment of the person’s eating disorder health literacy and stage of change *[9]*’.* Consideration should be given to the reality that evidence based and or best practice treatments may not have been identified, endorsed or available during a person’s illness, and treatment may not have been delivered in a way that was experienced as therapeutic and contributed to mis-trust, loss of hope and treatment disengagement.

Recently Austin et al. 2023 [134] proposed to unify diagnostic measures used in eating disorders research and clinical practice, via their international consensus. They endorse the Clinical Impairment Assessment, and the 12-item WHO Disability Assessment Schedule 2. In order to capture the impact of AN on self and identity within the quality of life rubric, an additional or custom instrument is required to fully capture the ‘global impoverishment’—for example Stanghellini et al.’s, Identity and Eating Disorders (IDEA) [124] 2012) or SCIM, Self Concept and Identity Measure [135]-See Additional file 1.

How SE-AN differs from other types of SEED—bulimia nervosa (BN) or binge eating disorder (BED) (i.e. SEED-BN and SEED-BED) is unknown [33]. The two refinements we have proposed to the SE-AN diagnostic lend to encompassing other enduring eating disorders via; (a) removal of underweight specifier (as BMI is a poor predictor of severity this is the only delineator of AN-BP with BN and Atypical AN so all can be encompassed) and; (b) including physical sequalae generally to align with those most related to AN or BN.

### Clinical implications: treatment considerations

We have discussed how shame, sensitivity, somatisation and dissociation may manifest in SE-AN and these features can be screened empirically to assist in collaborating with patients on treatment directions (see Additional file 1). A reduced opportunity to learn about and label sensations due to the physiological effects of AN, could be amenable to change at different time points representing a reversable legacy of the prolonged starved state via treatment. This is consistent with findings elsewhere that suggest that (i) there may be fixed associations between bodily feelings and emotional states [136] (ii) neuro-progressive changes [114] can occur secondary to the starvation and (iii) alexithymia may be a maladaptive consequence of shame and trauma [91].

The artist’s legacy invites clinicians to broaden clinical practice, to include alternative means of communicating their SE-AN experience, beyond verbal, cognitive and behavioural approaches. In this example, visual art is one alternative indicated as a powerful tool to communicate their experience. There is a notable paucity of research, despite systematic reviews indicating the feasibility of art in therapy [137, 138].This artist’s work was not formal or directed art therapy it was without supervision or comment on her art and to our knowledge she did not bring this artistic exploration to therapy. The professional artist freely depicted her journey and this is how we would distinguish between art as method, art as treatment and cannot say how this would translate for others.

Likewise, the somatic and dissociative aspects described in this study may lend to adaptations to clinical practice which include ‘body process’ or bottom-up therapeutic approaches [125, 139–141]. The acceptability and efficacy of this is unknown for SE-AN and care would need to be taken to respecting difficulties people with SE-AN may have in connecting to their bodies, felt sense of self (interoception) and emotions [125]. Further studies comparing measures of shame in EDs are indicated, given a range of instruments are used [92]. The internalised negative self-perceptions evident in this study could indicate internalised self-schema [53] internalised (sadistic) self-objects [142] and narrative incoherence [143]. As such these constructs could offer implications for new or existing therapies of such acknowledgement [144].

Hope is challenged by the material in this research. The dominant message that hope means ‘full recovery’ and return to ‘normality’ has potential to undermine what is available to a person. Whereas for the artist there were many layers of hope in her experience, including hope to be released from her pain and suffering through death. While holding hope of a full recovery for people with AN is paramount, especially knowing that it can occur after decades of illness [4], it needs to consider the full context of the person’s illness history. This study highlights the experience of one of the twenty people who die to AN, which raises questions about treatment considerations for the clinical reality of death as an outcome for some people [145]. It implores the field to:

1. consider what it is we are asking of people with SE-AN in relinquishing their illness, including our goals for wellness and definitions for recovery;
2. continue discussions about adaptations to care across the spectrum of illness experience, including end of life care for some; and
3. consider the impact of loss and grief to a person, in their course of SE-AN treatment.

The artist exemplified a search for meaning and to make peace with a life lived with AN through the acceptance of death and finds freedom in this. This manifests in the larger, more vibrantly coloured penultimate artworks as well as spiritual companionship with a deceased artist—connecting with her biography. This is a compelling example of the value in shifting therapeutic goals toward making meaning of a life lived [146] where premature death is imminent, compared with the dismissing a life lived with AN as one not worth living. The need for specialised expertise in grief and loss when working with enduring eating disorders is implicated, including the understanding that non-finite, ‘living’ losses impact the person’s identity and produce further uncertainty and loss of control [147].

### Future research opportunities

This study illuminates a range of research opportunities for SE-AN. The delineation of illness duration in all future studies of AN would assist in distinguishing SE-AN and developing consistent definitions will support synthesis of findings. The proposed putative defining features of SE-AN, include criteria related to severity, duration, and treatments. This study highlights limitations in these defining features, and consideration of self and identity disturbance, including dissociative and shame processes together with ‘global impoverishment’ are needed.

There is a need for further qualitative studies exploring the phenomenology of SE-AN, especially accounts of recovery. This may offer insight into a person-centered SE-AN definition of recovery, which likely involves negotiating a life lived with SE-AN. Future research could utilize cross case analysis with the artist’s account and new accounts could ultimately be included in a synthesis. Well-designed longitudinal studies are needed to clarify temporal processes related to illness persistence and to test various hypotheses related to treatment outcomes for example (i) the impact of exploratory psychotherapy concurrent to weight restoration (where the former is usually delayed until full weight restoration has occurred) (ii) treatment implications in the case of metabolic adaptations in SE-AN (iii) impact on outcomes of attending to the processes highlighted in this analysis (e.g. shame, dissociation) at an earlier stage of AN treatment (iv) temporal relationships and causal direction when considering progression of AN to SE-AN. In other-words, distinguishing those intra-psychic processes that predate AN, arise secondary to it and or are present at early stages of AN, those that arise later and those that become maintaining factors for SE-AN. If we consider these processes as having potential to undermine the development of self and a stable identity, they could be important early targets in treatment at different time points, prior to entrenchment, in support of personalized treatment approaches. This would also assist in refining the SE-AN diagnostic.

In considering a transdiagnostic view in SE-ED, it is unknown if ‘self’ is interrupted to the same extent in BN and other EDs or if it is more closely connected to the corporeal self, i.e. body image. Also, whether intra- psychic processes of the SE-AN experience i.e. somatization, shame, extremes of sensitivity are equally manifest in chronic cases of other EDs. In our synthesis of studies, which included atypical AN [17], we found no differences in phenomenological aspects between AN/atypical AN and others have found the same regarding severity [148]. To extend the transdiagnostic further, in the case of binge eating disorder (BED), corporeal body image disturbance is excluded entirely from the BED diagnostic and it is unknown if BED manifests with the same impact on self as a progression of the BED as we purport in AN. Therefore, an additionally important delineation to explore in future studies of enduring eating disorders, whereby it is unknown whether the progression of self-disturbance in AN is something characteristic across all SE-ED or unique to the SE-AN experience. This is an avenue for potential future research.

This study exemplifies the non-acceptance of feelings where they are somaticized and experienced as aversive and non-finite/pervasive. Thus, the ambiguity regarding the interactions of somatization, sensitivity, interoception, alexithymia, and embodiment need further refinement as they relate to SE-AN [48, 149]. Including an exploration of the function of disconnection and dissociative processes in people with SE-AN. There is scope for more research and evaluation of art therapy for eating disorders, which is known to form part of some treatment programs.

### Strengths and limitations

This study is a unique literature contribution. Typically, lived experience exploration is facilitated by a single, semi-structured interview that is a top-down approach where the interviewer poses questions in line with their interests, to a brief timeframe. In contrast, in this account, research materials were carefully chosen by the artist to curate her perspectives. Her experiences were portrayed without prompting, over several months, in what would become the end of her life. Despite treatment forming a substantial portion of the artist’s life, it is unknown if she painted about her treatment experiences. There was only one artwork encompassing treatment, (Image 9) selected by the artist to include in her book. It is possible that (a) the artist did not have access to or wish to paint during inpatient treatment (b) treatment experiences remained unprocessed and unexpressed (c) they were not considered important to the artist or (d) they did not wish to share those experiences, for example out of respect to her treatment providers.

Careful attention was paid to bracketing of other knowledge about the participant, (known to LK) however it has been said [88] that a pre-existing trusting relationship with a person experiencing an eating disorder may facilitate the depth of material shared, also adding an experiential dimension to the interpretation of findings by the analyser. This offers a probable strength to the narrative. All three authors (LK, JC, PH) were involved in thematic analysis by way of conferring multiple perspectives in the interpretation of the data. The analytic processes were completed over a lengthy period (> 18 months), which allowed for further distillation of insights. Regarding limitations, as the artist is deceased it was not possible to involve them in the member checking process of the research findings. The artist’s family were invited to comment on the themes, and all agreed that no amendments were indicated, based on this process. Further research is needed to determine transferability of findings beyond this single account.

## Conclusion

The picture of SE-AN revealed in the analysis, extends upon current conceptualizations and challenges the notion of ‘body image disturbance’ as reductionistic. Body image disturbance should be extended to constructs that encompass a global disturbance of the embodied ‘self’. Various processes are implicated in illness persistence, eliciting multiple opportunities for further research testing, including further exploration of interactions between sensitivity, interoception, alexithymia, somatization, dissociation, embodiment, and shame. Additional criteria for the severe and enduring stages of illness related to ‘global impoverishment’ and self and identity processes are proposed for consideration in the testing of putative defining features of SE-AN as for supporting assessment and informing novel treatment directions with patients. The existing measurement tools discussed are indicated for research and clinical practice to inform a comprehensive assessment of SE-AN beyond BMI and duration of illness.

In the absence of adequate research, clinicians are by necessity encouraged to manage SE-AN by adapting treatment approaches from outside those with the most research evidence that have been considered best practice, as these have failed. There is a need for transformative treatment approaches that are tailored to the needs of the person that may include themes such as those related to shame, loss, grief, body process, self-compassion and supporting integration of the self.

### Supplementary Information

**Additional file 1**. Box 1: identifying Shame, Dissociation, Sensitivity, Somatisation and Identity in SE-AN.

## Data Availability

Artist’s book available online: Home | Seeing Anorexia—www.seeinganorexia.com

## Abbreviations

AN: Anorexia nervosa
BED: Binge eating disorder
BMI: Body mass index
BN: Bulimia nervosa
CBT: Cognitive behaviour therapy
DSM-5: Diagnostic and statistical manual
ED: Eating disorder
FBT: Family based therapy
GAF: Global assessment of functioning
GT: Gestalt therapy
HSPS: Highly sensitive person scale
IDEA: Identity and eating disorders
IPA: Interpretative phenomenological analysis
ISS: Internalized shame scale
MANTRA: Maudsley therapy for adults with anorexia nervosa
MP: Margaret preston
RGB: Red, green, blue
SE: Severe and enduring
SE-AN: Severe and enduring anorexia nervosa
SE-ED: Severe and enduring eating disorders
SPS: Sensory processing sensitivity
WHODAS: World Health Organisation Disability Assessment Schedule
SCIM: Self concept and identity measure
