# Supplementary material for: Anorexia nervosa through the lens of a severe and enduring experience: ‘lost in a big world’
Source: J Eat Disord. 2024 Jan 22;12:12. doi: 10.1186/s40337-023-00953-2 (PMC10804804; doi:10.1186/s40337-023-00953-2)
Supplement: Supplementary file 1 — Additional file 1. Box 1: identifying Shame, Dissociation, Sensitivity, Somatisation and Identity in SE-AN. [file 40337_2023_953_MOESM1_ESM.docx]

**Box 1: identifying Shame, Dissociation, Sensitivity, Somatisation and Identity in SE-AN**

**Shame**

- ESS, Experience of Shame Scale (Andrews, Qian, & Valentine, 2002) or;
- ISS, Internalized Shame Scale (Cook, 1990)

**Dissociation**

- PDES, Psycho-form: Dissociative Experiences Scale (Bernstein 1986) or;
- DIS-Q0, Dissociation Questionnaire (Vanderlinden 1992) or;
- SDQ-20, Somatoform Dissociation Questionnaire (Nijenhuis et al., 1996)

**Identity**

- IDEA, Identity and Eating Disorders (Stanghellini et al. 2012) or;
- SCIM, Self concept and Identity Measure (Kaufman et al 2015)

**Somatization**

- PHQ-15, Patient Health Questionnaire (Kroenke et al 2002)

**Sensitivity**

- HSPS, Highly Sensitive Person Scale (Aron & Aron 1997)
